# A Case Report and Literature Review of Bilateral Cervical Chondrocutaneous Branchial Remnants

**DOI:** 10.1155/2023/8475270

**Published:** 2023-06-12

**Authors:** Yuehua He, Huiling Zhu, Hang Ji, Weining Huang, Zhongrong Liu

**Affiliations:** Department of Dermatology, The First Affiliated Hospital of Guangzhou Medical University, Guangzhou, China

## Abstract

Chondrocutaneous branchial remnants (CCBRs) are rare congenital heterotopic tissue formations originating from the first or second embryonic branchial arches. Clinically, CCBRs are characterized predominantly by unilateral and solitary cartilaginous nodules found on the lower neck region. Herein, we present a case of CCBRs in a 9-year-old male patient who presented with horn-shaped projecting masses on either side of the anterior border of the sternocleidomastoid muscle. The pathological report following surgical resection revealed that the lesion was located in the dermis and consisted primarily of hyaline cartilage tissue enclosed by a fibrous capsule, with few local vascular proliferations. Based on the clinical and pathological features, the patient was ultimately diagnosed with congenital bilateral cervical chondrocutaneous branchial remnants.

## 1. Introduction

Chondrocutaneous branchial remnants are uncommon congenital cervical masses that typically manifest as unilateral, solitary hemispherical, horn-like, or papillary nodules. While first reported by Birkett in 1858 [1], they were also referred to as preauricular appendage, accessory tragus, accessory auricle, and warts before Atlan introduced the current term “chondrocutaneous branchial remnants” in 1997 [2, 3]. Chondrocutaneous branchial remnants may occur alone or in conjunction with other systemic abnormalities, affecting various bodily systems such as the visual, auditory, respiratory, gastrointestinal, genitourinary, cardiovascular, and musculoskeletal systems [3, 4]. The ideal approach for treating chondrocutaneous branchial remnants involves selecting the appropriate age for surgical excision of the lesion. Nevertheless, prior to surgery, it is critical to conduct the necessary preoperative examinations and ensure that the patient's psychological well-being is not adversely impacted. Herein, we present a rare case of bilateral cervical chondrocutaneous branchial remnants located on the sternocleidomastoid muscle, along with a comprehensive review of the existing literature on this condition.

## 2. Case

**Figure 1:**
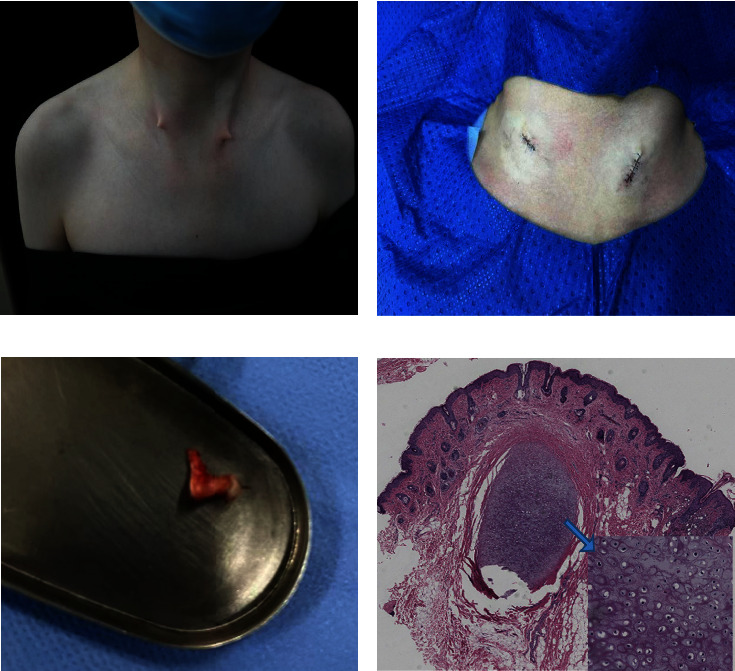
(a) Bilateral cervical branchial chondrocutaneous remnants in our patient prior to surgery. (b) The CCBRs were excised, and the skin was cosmetically sutured. (c) Gross appearance of the surgically excised mass. (d) The histopathological appearance of the excised lesion reveals a hyaline cartilage core.

Examination of a 9-year-old male patient at our outpatient department revealed painless masses that were protruding bilaterally on the anterior border of the sternocleidomastoid muscle in the neck, with no signs of infection or exudate detected upon palpation (Figure 1(a)). According to his mother, the lesions have been present since birth and have remained largely unchanged over time. During the outpatient visit, the child's parents told the doctor that the child had normal hearing since childhood. The family's advice was followed and no hearing test was carried out in the ENT department. The child came to our department seeking treatment for the masses which he considers a cosmetic hindrance. He had no previous medical history, and there were no reports of a similar condition in the family. Moreover, the child's mother had an uneventful pregnancy.

The physical examination of the patient did not reveal any significant findings. Besides, the chest X-ray showed no anomalies in the heart, lungs, or diaphragm. The child had not had any cardiac problems since childhood and the chest X-ray did not show any abnormalities in the size, shape, or position of the heart. Therefore, no further echocardiography was performed. A preoperative ultrasound examination of the neck revealed bilateral hypoechoic nodules in the subcutaneous region of the anterior cervical area. The left lesions were approximately 7 mm × 4 mm × 4 mm in size, while the right nodules were around 5 mm × 3 mm × 3 mm in size. They exhibited clear borders and irregular morphology, and no internal blood flow signal was detected.

Eventually, the child underwent surgery under general anesthesia. The bilateral horn-like masses were meticulously separated and excised, as shown in Figure 1(b). The mass resembled a core cartilage encapsulated by normal skin (Figure 1(c)). During the surgical intervention, no fistula was found in the neck.

Histopathological examination revealed that the lesion was located in the dermis and consisted primarily of hyaline cartilage tissue enclosed by a fibrous capsule with few local vascular proliferations (Figure 1(d)).

Considering the patient's history and clinical presentation, he was diagnosed with congenital bilateral cervical chondrocutaneous branchial remnants. No further treatment was thus given, and the patient was followed up regularly. To date, no signs of recurrence or hyperplasia have been observed.

## 3. Discussion

The nomenclature, differential diagnosis, etiology, possible genetic and molecular mechanisms, histopathology, comorbidities, investigative approach, treatment, and management recommendations for CCBRs are further discussed as follows.

### 3.1. Nomenclature

Birkett was the first to report a benign nodule formation in the neck that resembled an accessory auricle back in 1858. Since then, the terminology used to describe similar features has varied significantly, with various terms such as cutaneous appendages, malformations, papillomas, fibromas, warts, neck remnants, accessory tragus, and accessory auricle being utilized [5–9]. In 1997, Atlan et al. analyzed similar cases recorded over a 13-year period at his hospital and renamed this condition to chondrocutaneous branchial remnants based on the onset, embryonic origin, histological features, and complications related to this disease [3]. In 2014, Chander et al. reclassified this condition under the term chondrochondroma. He surmised that these lesions originated from pluripotent cells of the head and neck that proliferated and differentiated into cartilaginous tissues, given that the histological features were similar to those of chondrochondroma [10]. However, the pathological terminology was not well accepted worldwide.

The lesion was located at the anterior border of the sternocleidomastoid muscle in the neck and was consistent with the clinical presentation and histopathology of the chondrocutaneous branchial remnants, which was finally diagnosed in this child.

### 3.2. Differential Diagnosis

As a result of clinicians' failure to identify the histological characteristics of patients and their subjective diagnosis, chondrocutaneous branchial remnants have previously been referred to under a variety of terms.

The differential diagnosis of CCBRs for skin protruding formations includes warts, fibroids, polyps, epidermal-like cysts, and cervical follicular nevi [11]. Meanwhile, nonprotruding lesions include thymic cyst, thyroglossal duct cyst, branchial cleft cyst, pilomatrixoma, or hamartoma [12]. Common neck tumors in newborns, such as teratoma, embryoma, hamartoma, dermoid cyst, midline cyst, and sinus tract, should also be excluded [13]. Therefore, it is essential to conduct a complete physical examination, imaging, and histopathological analysis of lesions to identify patients with CCBRs.

### 3.3. Etiology

In 1997, Atlan et al. first proposed a mechanism for chondrocutaneous branchial remnants occurrence during external ear development. He hypothesized that the auricle is formed by the migration of the six auricular hillocks of the first and second branchial arches along the sternocleidomastoid muscle from the cervical to the facial region. During migration, part of the malleoli may be left behind, remaining in the neck and developing into a chondrocutaneous branchial remnant. As a result, CCBRs tend to be found around the sternocleidomastoid muscle and contain cartilaginous tissue similar to that found in the auricle. The authors further proposed that CCBRs are most likely to form in the neck's middle and lower third sections and that the cartilaginous remnants most likely originate from the second or posterior branchial arch [3]. On the other hand, Lee et al. previously surmised that CCBRs formation results from incomplete occlusion of the gill organ, causing cartilage-differentiating cells to remain in the neck during embryonic migration [9]. Based on Sperling's histological classification, the presence of elastic cartilage was indicative that chondrocutaneous branchial remnants originated from the auricle and the first or second branchial arch, while the presence of hyaline cartilage ruled out an auricle origin, suggesting that it came from the second or lower branchial arch [14]. Begovic et al. previously inferred that CCBRs most likely originate from the first and second branchial arches based on the presence of both elastic and hyaline cartilages in their collected specimens [15].

Herein, the histological analysis of our patient revealed that the excised specimen consisted of hyaline cartilage, pointing towards a second branchial arch or inferior branchial arch origin. Nonetheless, further in-depth studies are warranted to substantiate and clarify the intricate etiology of CCBRs.

### 3.4. Genetic-Molecular Mechanisms

Pham Dang et al. previously reported on five individuals with familial chondrocutaneous branchial remnants over three generations, indicating that this condition might also be hereditary in an autosomal dominant manner and is not significantly related to gender [16]. However, Ishigaki et al.'s case series had more male than female patients, suggesting that males are more susceptible to CCBRs [2, 3]. Given that accessory auricular displays a similar cartilaginous tissue structure to CCBRs, we further elaborated on its genetic features. Of the 502 patients with accessory auricles reported by Hwang et al., 11% had a familial history [17]. Teja and Cooper reported on a family in which both the brother and sister had accessory tragus [18], while Bendet reported on a family with four generations of accessory tragus [19]. Yang et al. [20] analyzed the genetic mapping of 11 families with autosomal dominant accessory auricular anomaly (ADAAA) and found that accessory auricle was an autosomal dominant trait, inherited from male to male offspring, and the locus was localized on chromosome 14 at 14q11.2-q12. Taken together, the abovementioned findings suggest that genetics may be a factor in the formation of the accessory auricle. Therefore, it is important to question the family history during medical history taking. The absence of CCBRs in the family history of our patient indicates that factors other than genetics may also be involved in the development of this condition. For instance, Keer et al. previously uncovered that Mcrs1 is essential for the development of the branchial arch and cranial cartilage [21]. Therefore, it could be likely that abnormal regulation of Mcrs1 may be associated with CCBRs, but further study is required for validation.

### 3.5. Histopathology

Chondrocutaneous branchial remnants are typically characterized by a core of elastic or hyaline cartilage located in the deep dermis or subcutaneous fat layer, surrounded by a fibrous capsule [2, 9, 15]. In some patients, there may also be hair follicles, sebaceous glands, sweat glands, and ectopic fat tissue, as well as components such as cartilage, skeletal muscle, neuromuscular tissue, nerve bundles, and circular corpuscles [22].

### 3.6. Comorbidities

In 1997, Atlan et al. reported that chondrocutaneous branchial remnants were associated with several abnormalities that could affect the auditory (neurosensory deafness, plagiocephaly, and external ear malformations), respiratory (subluxation of the spoon cartilage, tracheal softening, and pulmonary atelectasis), orogastric (cleft palate, ligature, oronasal reflux, and inguinal hernia), genitourinary (hydronephrosis, hypospadias, and cryptorchidism), cardiovascular (atrial septal defect, ventricular septal defect, and patent ductus arteriosus), musculoskeletal (deformed feet and congenital hip dislocation), and visual systems (strabismus), as well as induce Parkinson's disease [3]. Among the 29 CCBR cases reported by Ishigaki et al. (19 males and 10 females), 8 patients had comorbidities, 1 female patient had unilateral facial paralysis, and the remaining 7 male patients had: 2 cases of penis palmatus, 1 case of bilateral undescended testis, 1 case of umbilical hernia, 1 case of internal strabismus in the right eye, 1 case of soft meningeal cyst, and 1 case of left cervical cyst combined with nodular tongue [2]. The study shows that the incidence of CCBRs is higher in men than in women, as were the comorbidities. Of the 17 patients (10 males and 7 females) reported by Begovic et al., five had complications, the three male patients had Branchio-oto-renal syndrome, vesicoureteral reflux, and ventricular septal defect, respectively, while the 2 females had sinus preauricularis, atrial septal defect, and ventricular septal defect [15]. Meanwhile, Woo and Kim reported on three CCBRs patients that developed congenital thyroid hemiagenesis, subependymal cyst, and conjunctiva [23]. The Japanese scholar Kono et al. reported a female child born with cartilaginous cutaneous gill remnants combined with a right cheek and a right preauricular screen parapleural and also reviewed the literature and found five children diagnosed with cartilaginous cutaneous gill remnants combined with a facial or preauricular screen parapleural [24]. Interestingly, another study found that a 25-year-old female diagnosed with chondrocutaneous branchial remnants had previously untreated Meniere's disease and presented with no other discomfort [12].

Chondrocutaneous branchial remnants can lead to various systemic diseases and complications. These reports highlight the importance of systematic physical examination and ancillary tests during patient consultation to identify comorbidities that may impact treatment decisions and outcomes.

### 3.7. Investigations

Investigations are crucial for the timely detection and management of CCBRs. Gilboa et al. identified chondrocutaneous branchial remnants in three of 51,343 fetuses during early routine ultrasound examinations of pregnant women. In the first case, termination was chosen by the parents, while the second patient underwent postnatal surgical excision. The third fetus had cardiac abnormalities, and after careful consideration, termination was performed [4]. This shows that early screening during pregnancy can help reduce the number of children born with severe karyotypic defects.

In addition, it is important for patients with CCBRs to undergo a thorough physical examination. For CCBRs identified after birth, it is essential to monitor their growth and development. Moreover, neuropsychiatric, vision, hearing, maxillofacial, genitourinary, respiratory, and cardiac examinations must be conducted regularly. In addition, renal function, ECG, chest and abdominal ultrasounds, X-rays, and electroencephalograms must also be performed. Importantly, an ultrasound examination of the mass is necessary to prepare the patient for surgery and avoid intraoperative injury to surrounding blood vessels, nerves, tissues, and organs.

### 3.8. Treatment and Recommendations

When the diagnosis of chondrocutaneous branchial remnants is straightforward, surgical removal under anesthesia is typically the treatment of choice. However, in cases with additional complications or comorbidities, symptomatic management may be necessary prior to surgery to reduce the risk of damage to surrounding tissues and organs. The type of anesthesia used will typically depend on the patient's compliance.

Since chondrocutaneous branchial remnants are often present at birth, it is important for families to seek medical attention promptly to avoid complications and psychological distress later on. Notably, early intervention before the child is enrolled at school can help reduce the risk of surgical complications and prevent social or psychological issues.

## 4. Conclusion

The diagnosis and treatment of chondrocutaneous branchial remnants are of utmost importance to prevent complications and improve patient outcomes. While a small proportion of patients may present with comorbidities or complications, most patients are asymptomatic. Therefore, a comprehensive history, physical examination, and appropriate ancillary tests are crucial in making an accurate diagnosis. Early intervention through surgical excision is recommended to avoid any potential psychological effects on the child. In addition, conducting renal function, ECG, echocardiography, audiology, and imaging can help identify any associated abnormalities and enable effective treatment planning. Taking a proactive approach and providing timely and appropriate care can significantly improve the quality of life for patients with chondrocutaneous branchial remnants.
